# Do Novel Biomarkers Have Utility in the Diagnosis and Prognosis of AKI? PRO

**DOI:** 10.34067/KID.0000000000000189

**Published:** 2023-06-08

**Authors:** John A. Kellum, Stuart L. Goldstein

**Affiliations:** 1 Department of Critical Care Medicine, University of Pittsburgh, Pittsburgh, Pennsylvania; 2 Cincinnati Children's Hospital Medical Center, Cinncinnati, Ohio; 3 Department of Pediatrics, University of Cincinnati College of Medicine, Cincinnati, Ohio

**Keywords:** kidney damage, kidney disfunction, *in vitro* diagnostics, neutrophil gelatinase-associated lipocalin, NGAL, tissue inhibitor of metalloproteinases-2, TIMP-2, insulin-like growth factor binding protein 7, IGFBP7

This article will present the pro-side of debate concerning the utility of novel biomarkers in the diagnosis and prognosis of AKI.

## Diagnosis of AKI

Examination of the utility of novel biomarkers for AKI diagnosis and prognosis should begin with a discussion of AKI clinical assessment without these tools. Currently, AKI is defined and staged by rapid changes in serum creatinine (SCr) or urine output (UOP).^1^ These biomarkers are well known to clinicians and relatively easy to measure. SCr is also used extensively for kidney function assessment in patients with CKD. Unfortunately, SCr has many well-documented limitations in kidney function assessment in the acute state and cannot directly assess kidney damage nor does it distinguish acute from chronic dysfunction.

UOP has some advantages as an acute marker because changes in urine flow may occur rapidly compared with SCr (minutes versus hours). However, UOP is neither specific to kidney injury nor highly sensitive since forms of nonoliguric AKI (*e.g.*, nephrotoxicity) are common. The combination of SCr and UOP provide more information than either marker alone,^2,3^ but still, these traditional measures of kidney function are inadequate for assessing AKI. Clinicians, therefore, use a variety of additional tools to evaluate possible cases of AKI. These include imaging, urine chemistry, and urine microscopy.

The presumed gold standard for AKI diagnosis is kidney biopsy, but this procedure is performed too infrequently to compare it with other diagnostic tests. When performed, the evidence of acute tubular injury is usually found in patients meeting Kidney Disease Improving Global Outcomes (KDIGO) AKI stage 2 or 3 criteria. Yet, patients without evidence of kidney disease do not undergo biopsy, so it is difficult to estimate the proportion of patients with kidney damage missed by relying on these crude functional criteria. Experimental animal AKI models that cause extensive evidence of damage on histologic examination may produce only mild or transient dysfunction. AKI often results in patchy injury with some tubules severely affected and others spared.^4^

## Damage versus Dysfunction

With kidney damage difficult to measure, and with dysfunction an unreliable surrogate for damage, it is no surprise that clinicians have difficulty diagnosing AKI. Decreased kidney function measured by increased SCr or decreased UOP may also occur without kidney damage, for example, in the setting of hypovolemia. In this setting, AKI may be purely “functional.” Unfortunately, ancillary tests (*e.g.*, imaging) are insensitive to kidney damage and may seem normal despite injury. Sometimes kidney damage can be inferred from persistent functional impairment, but the absence of persistent dysfunction does not exclude irreversible loss of nephrons because of substantial reserve capacity. Detecting kidney damage in settings where function is normal or only mildly or transiently impaired is critical because kidneys have limited capacity to regenerate. Once fibrosis has occurred, those nephrons are lost. Conversely, determining that dysfunction is not related to damage can guide management. Some AKI biomarkers were discovered by inducing kidney injury in animals, and they have subsequently been established to detect damage in different parts of the human kidney.^5^

## Time

Another critical limitation of SCr is that it may take up to 24 hours to indicate a significant change in function. UOP may change faster, but it may not change at all, and when it does, it still takes some time to assess—*i.e.*, it must be measured over at least 6 hours to determine whether it is reduced. Novel biomarkers are considerably faster. Tissue inhibitor of metalloproteinases-2 (TIMP-2) and insulin-like growth factor-binding protein 7 (IGFBP7) are preformed molecules that are released into the urine within minutes of injury.^6^ Similarly, neutrophil gelatinase-associated lipocalin (NGAL) is upregulated and observed in the urine at 2 hours, 36 hours before changes in SCr for patients with AKI.^7^

Failure to detect kidney damage promptly may reduce the ability to reverse it. Existing management strategies for AKI include discontinuing offending drugs, relieving obstruction, and optimization of hemodynamics.^1^ Biomarker-guided implementation of a care bundle on the basis of the KDIGO guideline demonstrated a significant reduction in AKI events^8,9^ after cardiac surgery, and these studies have also shown that these interventions are not applied routinely in patients without AKI, so the biomarker has a material effect on patient care.

## Prognosis

AKI negatively affects short-term and long-term survival. In adults, mortality in the year after AKI exceeds 25%, and the rate of progression of or to CKD exceeds 20%.^10^ Furthermore, AKI recures in nearly a third of patients in the year after an episode. Although not developed for this purpose, novel AKI biomarkers provide additional prognostic information over and above KDIGO-based AKI staging. Importantly, death or dialysis rates by nine months^11,12^ or by hospital discharge^13^ are higher in patients with increased TIMP-2•IGFBP7 only when functional changes have also occurred. As such, the prognostic signal would seem to be specific to AKI and not a “general” mortality indicator. The results are also consistent with the nature of TIMP-2 and IGFBP7 as stress rather than damage biomarkers. Stress in isolation may be benign, but when it coexists with dysfunction, it may signal actual damage. However, this interpretation is challenged by the work of Husain-Syed and colleagues who have demonstrated that increased urinary TIMP-2•IGFBP7 after cardiac surgery is associated with loss of renal functional reserve at three months even in the absence of AKI.^14^ It may be that crude measures, such as death or dialysis, do not change when kidney stress occurs without dysfunction, but measuring renal functional reserve may reveal that this stress resulted in kidney damage.

Given the potential for novel biomarkers to provide information beyond KDIGO staging, the 23rd Acute Disease Quality Initiative workgroup proposed an expanded classification for AKI adding biomarkers for each stage (Table 1).^15^ The first validation of this classification has been recently published using TIMP-2•IGFBP7 in adults with sepsis. In patients who developed AKI using standard functional criteria (SCr and UOP) according to KDIGO staging, the addition of urinary TIMP-2•IGFBP7 >2.0 (ng/ml)^2^/1000 identified patients with lower 30-day survival within the functional stages. Furthermore, TIMP-2•IGFBP7 >1.0 (ng/ml)^2^/1000 may be helpful in stratifying patients in the absence of functional criteria for AKI.

**Table 1:** Proposed new definition and staging of AKI by the 23rd Acute Disease Quality Initiative consensus conference

KDIGO Stages	Functional Criteria	Biomarkers	New Stages
No AKI	No increased sCr ≥0.3 mg/dl in ≤48 h AND no increased sCr ≥1.5 from baseline in 7 d AND UO >0.5 ml/kg per hour in 6 h period	**−**	No AKI
		**+**	Stage 1S
Stage 1	Increased sCr ≥0.3 mg/dl in ≤48 h OR increased sCr 1.5–1.9 times from baseline in <7 d OR UO <0.5 ml/kg per hour for 6–12 h	**−**	Stage 1A
		**+**	Stage 1B
Stage 2	Increased sCr 2.0–2.9 times from baseline OR UO <0.5 ml/kg per hour for ≥12 h	**−**	Stage 2A
		**+**	Stage 2B
Stage 3	Increased sCr ≥3.0 times from baseline OR sCr ≥4.0 mg/dl with acute increase of ≥0.3 mg/dl OR UO <0.3 ml/kg per hour for ≥24 h OR anuria for ≥12 h OR initiation of renal replacement therapy	**−**	Stage 3A
		**+**	Stage 3B

KDIGO, Kidney Disease: Improving Global Outcomes; sCr, serum creatinine; UO, urinary output.

Adapted from Acute Disease Quality Initiative 23 (Figure 5) on www.ADQI.org and published by Ostermann and colleagues.^15^

Systematic reviews of NGAL in adults and children similarly demonstrate worse outcomes (mortality and hospital length of stay) in patients with both functional and structural AKI.^16,17^ Although mortality rates for critically ill children are lower than those for adults, NGAL has been studied extensively to delineate functional *vs* structural *vs.* combined in children after cardiac surgery and sepsis,^18,19^ also showing worse outcomes for patients with structural or combined structural and functional AKI.

## Clinical Experience

It is not known what proportion of physicians use novel AKI biomarkers in their clinical practice, but estimates are quite low—even among those with a critical care nephrology focus.^20^ TIMP-2 and IGFBP7 were incorporated into a clinical laboratory test, the NephroCheck test, which received US Food and Drug Administration approval in 2014.^21^ NGAL use was first reported clinically in 2017.^22^ We use these tests daily in our clinical practice and find utility in them. Most arguments against their use do not reflect experience and instead challenge whether they are accurate enough despite multiple meta-analyses concluding acceptable operating characteristics or are concerned about limitations when CKD or diverse etiologies of AKI are present.^23^ However, biomarkers, such as TIMP-2•IGFBP7 and NGAL, have been shown to perform quite well in patients with CKD^24,25^ or when the etiology of AKI is sepsis,^19,26^ surgery,^27,28^ or nephrotoxins.^29,30^ Some clinicians have wondered whether interventions can be effective or even whether all available are already being applied. However, the intent of diagnostic and prognostic testing is to bring personalized medicine to the bedside. One size does not fit all for AKI any more than for other illnesses. Consensus across subspecialties exists for personalized care in response to biomarkers,^31^ and various experts have already included biomarkers in standardized AKI management guidelines for cardiac surgery.^32^ Others are likely to follow.

Finally, many authors seem confused by how the costs of diagnostic tests fit into the health care landscape and who pays for them.^23^ Novel AKI biomarkers are quite inexpensive in comparison with many other routine tests, including imaging and electrophysiologic testing. Of course, they are currently more expensive than creatinine, but any new test will cost more relative to established clinical laboratory assays. In all cases, the cost of AKI itself, estimated to be in thousands to tens of thousands,^33^ should be considered when evaluating the costs of tools used to prevent it.

It is thus our opinion, as clinicians, scientists, and AKI experts, that novel biomarkers, such as NGAL and TIMP-2•IGFBP7, have considerable utility in the diagnosis and prognosis of AKI. This judgment is based on an extensive literature base including our own studies but also our own extensive clinical experience.

## References

[1] KDIGO Clinical practice guideline for acute kidney injury. Kidney Int. 2012;2(2):1–141.

[2] Kellum JA Sileanu FE Murugan R Lucko N Shaw AD Clermont G. Classifying AKI by urine output versus serum creatinine level. J Am Soc Nephrol. 2015;26(9):2231–2238. doi:10.1681/ASN.2014070724 25568178 PMC4552117

[3] Kaddourah A Basu RK Goldstein SL Sutherland SM. Oliguria and acute kidney injury in critically ill children: implications for diagnosis and outcomes. Pediatr Crit Care Med. 2019;20(4):332–339. doi:10.1097/pcc.0000000000001866 30676490

[4] Chawla LS Eggers PW Star RA Kimmel PL. Acute kidney injury and chronic kidney disease as interconnected syndromes. N Engl J Med. 2014;371(1):58–66. doi:10.1056/nejmra1214243 24988558 PMC9720902

[5] Alge JL Arthur JM. Biomarkers of AKI: a review of mechanistic relevance and potential therapeutic implications. Clin J Am Soc Nephrol. 2015;10(1):147–155. doi:10.2215/CJN.12191213 25092601 PMC4284423

[6] Johnson ACM Zager RA. Mechanisms underlying increased TIMP2 and IGFBP7 urinary excretion in experimental AKI. J Am Soc Nephrol. 2018;29(8):2157–2167. doi:10.1681/ASN.2018030265 29980651 PMC6065093

[7] Mishra J Dent C Tarabishi R, . Neutrophil gelatinase-associated lipocalin (NGAL) as a biomarker for acute renal injury after cardiac surgery. Lancet. 2005;365(9466):1231–1238. doi:10.1016/s0140-6736(05)74811-x 15811456

[8] Meersch M Schmidt C Hoffmeier A, . Prevention of cardiac surgery-associated AKI by implementing the KDIGO guidelines in high risk patients identified by biomarkers: the PrevAKI randomized controlled trial. Intensive Care Med. 2017;43(11):1551–1561. doi:10.1007/s00134-016-4670-3 28110412 PMC5633630

[9] Zarbock A Kullmar M Ostermann M, . Prevention of cardiac surgery-associated acute kidney injury by implementing the KDIGO guidelines in high-risk patients identified by biomarkers: the PrevAKI-multicenter randomized controlled trial. Anesth Analg. 2021;133(2):292–302. doi:10.1213/ane.0000000000005458 33684086

[10] James MT Bhatt M Pannu N Tonelli M. Long-term outcomes of acute kidney injury and strategies for improved care. Nat Rev Nephrol. 2020;16(4):193–205. doi:10.1038/s41581-019-0247-z 32051567

[11] Joannidis M Forni LG Haase M, . Use of cell cycle arrest biomarkers in conjunction with classical markers of acute kidney injury. Crit Care Med. 2019;47(10):e820–e826. doi:10.1097/ccm.0000000000003907 31343478 PMC6750148

[12] Koyner JL Shaw AD Chawla LS, . Tissue inhibitor metalloproteinase-2 (TIMP-2) • IGF-Binding protein-7 (IGFBP7) levels are associated with adverse long-term outcomes in patients with AKI. J Am Soc Nephrol. 2015;26(7):1747–1754. doi:10.1681/ASN.2014060556 25535301 PMC4483589

[13] Xie Y Ankawi G Yang B, . Tissue inhibitor metalloproteinase-2 (TIMP-2) • IGF-binding protein-7 (IGFBP7) levels are associated with adverse outcomes in patients in the intensive care unit with acute kidney injury. Kidney Int. 2019;95(6):1486–1493. doi:10.1016/j.kint.2019.01.020 30982674

[14] Husain-Syed F Ferrari F Sharma A, . Persistent decrease of renal functional reserve in patients after cardiac surgery-associated acute kidney injury despite clinical recovery. Nephrol Dial Transplant. 2019;34(2):308–317. doi:10.1093/ndt/gfy227 30053231

[15] Ostermann M Zarbock A Goldstein S, . Recommendations on acute kidney injury biomarkers from the acute disease quality initiative consensus conference: a consensus statement. JAMA Netw Open. 2020;3(10):e2019209. doi:10.1001/jamanetworkopen.2020.19209 33021646

[16] Haase M Bellomo R Devarajan P Schlattmann P Haase-Fielitz A. Accuracy of neutrophil gelatinase-associated lipocalin (NGAL) in diagnosis and prognosis in acute kidney injury: a systematic review and meta-analysis. Am J Kidney Dis. 2009;54(6):1012–1024. doi:10.1053/j.ajkd.2009.07.020 19850388

[17] Haase M Devarajan P Haase-Fielitz A, . The outcome of neutrophil gelatinase-associated lipocalin-positive subclinical acute kidney injury: a multicenter pooled analysis of prospective studies. J Am Coll Cardiol. 2011;57(17):1752–1761. doi:10.1016/j.jacc.2010.11.051 21511111 PMC4866647

[18] Basu RK Wong HR Krawczeski CD, . Combining functional and tubular damage biomarkers improves diagnostic precision for acute kidney injury after cardiac surgery. J Am Coll Cardiol. 2014;64(25):2753–2762. doi:10.1016/j.jacc.2014.09.066 25541128 PMC4310455

[19] Stanski N Menon S Goldstein SL Basu RK. Integration of urinary neutrophil gelatinase-associated lipocalin with serum creatinine delineates acute kidney injury phenotypes in critically ill children. J Crit Care. 2019;53:1–7. doi:10.1016/j.jcrc.2019.05.017 31174170

[20] Digvijay K Neri M Fan W Ricci Z Ronco C. International survey on the management of acute kidney injury and continuous renal replacement therapies: year 2018. Blood Purif. 2019;47(1-3):113–119. doi:10.1159/000493724 30269144

[21] Food and Drug Administration. Nephrocheck Test System; 2014.

[22] Varnell CD Jr. Goldstein SL Devarajan P Basu RK. Impact of near real-time urine neutrophil gelatinase-associated lipocalin assessment on clinical practice. Kidney Int Rep. 2017;2(6):1243–1249. doi:10.1016/j.ekir.2017.05.012 29270534 PMC5733822

[23] Claure-Del Granado R Macedo E Chavez-Iniguez JS. Biomarkers for early diagnosis of AKI: could it backfire? Kidney360. 2022;3(10):1780–1784. doi:10.34067/kid.0001012022 36514722 PMC9717653

[24] Heung M Ortega LM Chawla LS, . Common chronic conditions do not affect performance of cell cycle arrest biomarkers for risk stratification of acute kidney injury. Nephrol Dial Transplant. 2016;31(10):1633–1640. doi:10.1093/ndt/gfw241 27342582 PMC5039344

[25] Guo L Zhao Y Yong Z Zhao W. Evaluation value of neutrophil gelatinase-associated lipocalin for the renal dysfunction of patients with chronic kidney disease: a meta-analysis. Aging Med (Milton). 2018;1(2):185–196. doi:10.1002/agm2.12033 31942496 PMC6880667

[26] Honore PM Nguyen HB Gong M, . Urinary tissue inhibitor of metalloproteinase-2 and insulin-like growth factor-binding protein 7 for risk stratification of acute kidney injury in patients with sepsis. Crit Care Med. 2016;44(10):1851–1860. doi:10.1097/ccm.0000000000001827 27355527 PMC5089124

[27] Gunnerson KJ Shaw AD Chawla LS, . TIMP2*IGFBP7 biomarker panel accurately predicts acute kidney injury in high-risk surgical patients. J Trauma Acute Care Surg. 2016;80(2):243–249. doi:10.1097/ta.0000000000000912 26816218 PMC4729326

[28] Krawczeski CD Goldstein SL Woo JG, . Temporal relationship and predictive value of urinary acute kidney injury biomarkers after pediatric cardiopulmonary bypass. J Am Coll Cardiol. 2011;58(22):2301–2309. doi:10.1016/j.jacc.2011.08.017 22093507 PMC3220882

[29] Ostermann M McCullough PA Forni LG, . Kinetics of urinary cell cycle arrest markers for acute kidney injury following exposure to potential renal insults. Crit Care Med. 2018;46(3):375–383. doi:10.1097/ccm.0000000000002847 29189343 PMC5821475

[30] Goldstein SL Krallman KA Schmerge A, . Urinary neutrophil gelatinase-associated lipocalin rules out nephrotoxic acute kidney injury in children. Pediatr Nephrol. 2021;36(7):1915–1921. doi:10.1007/s00467-020-04898-5 33459927

[31] Guzzi LM Bergler T Binnall B, . Clinical use of [TIMP-2]*[IGFBP7] biomarker testing to assess risk of acute kidney injury in critical care: guidance from an expert panel. Crit Care. 2019;23(1):225. doi:10.1186/s13054-019-2504-8 31221200 PMC6585126

[32] Milne B Gilbey T Kunst G. Perioperative management of the patient at high-risk for cardiac surgery-associated acute kidney injury. J Cardiothorac Vasc Anesth. 2022;36(12):4460–4482. doi:10.1053/j.jvca.2022.08.016 36241503

[33] Amin AP McNeely C Spertus JA, . Incremental cost of acute kidney injury after percutaneous coronary intervention in the United States. Am J Cardiol. 2020;125(1):29–33. doi:10.1016/j.amjcard.2019.09.042 31711633

